# Light-Mediated Signaling and Metabolic Changes Coordinate Stomatal Opening and Closure

**DOI:** 10.3389/fpls.2020.601478

**Published:** 2020-12-04

**Authors:** Juan Yang, Chunlian Li, Dexin Kong, Fangyan Guo, Hongbin Wei

**Affiliations:** ^1^College of Life Sciences, State Key Laboratory for Conservation and Utilization of Subtropical Agro-Bioresources, South China Agricultural University, Guangzhou, China; ^2^School of Life Sciences, Southwest University, Chongqing, China

**Keywords:** light signaling, stomatal movement, negative mechanism, trade-off, *Arabidopsis thaliana*, guard cell metabolism

## Abstract

Stomata are valves on the leaf surface controlling carbon dioxide (CO_2_) influx for photosynthesis and water loss by transpiration. Thus, plants have to evolve elaborate mechanisms controlling stomatal aperture to allow efficient photosynthesis while avoid excessive water loss. Light is not only the energy source for photosynthesis but also an important signal regulating stomatal movement during dark-to-light transition. Our knowledge concerning blue and red light signaling and light-induced metabolite changes that contribute to stomatal opening are accumulating. This review summarizes recent advances on the signaling components that lie between the perception of blue/red light and activation of the PM H^+^-ATPases, and on the negative regulation of stomatal opening by red light-activated phyB signaling and ultraviolet (UV-B and UV-A) irradiation. Besides, light-regulated guard cell (GC)-specific metabolic levels, mesophyll-derived sucrose, and CO_2_ concentration within GCs also play dual roles in stomatal opening. Thus, light-induced stomatal opening is tightly accompanied by brake mechanisms, allowing plants to coordinate carbon gain and water loss. Knowledge on the mechanisms regulating the trade-off between stomatal opening and closure may have potential applications toward generating superior crops with improved water use efficiency (CO_2_ gain vs. water loss).

## Introduction

Stomata are leaf epidermal structures comprised of two guard cells (GCs) surrounding a pore, with GCs flanked by two lateral subsidiary cells in some species. Stomata act as valves on the leaf surface, simultaneously controlling carbon dioxide (CO_2_) uptake for photosynthesis and water loss by transpiration. The swelling (increased turgor due to water influx) of GCs confers stomatal opening, while the shrinkage (decreased turgor due to water efflux) of GCs causes stomatal closure. Changes in the osmolyte content of GCs and stomatal aperture are governed by internal cues (e.g., circadian clock), biotic signals (Ye and Murata, 2016; Melotto et al., 2017), and diverse environmental factors, including light, CO_2_ concentration, water deficit, and temperature (Shimazaki et al., 2007; Mott et al., 2008; Chen et al., 2012; Lawson and Blatt, 2014; Kostaki et al., 2020). Stomatal conductance (*g_s_*) is a measure of the degree of stomatal opening, reflecting the capacity of stomata to exchange CO_2_ and water vapor with the environment. It is well established that high *g_s_* is correlated with higher rates of photosynthesis (*A*), while low *g_s_* restrains CO_2_ diffusion and *A*, resulting in hampered biomass and yield (Lawson et al., 2012; Matthews et al., 2017). It is noted that high *g_s_* may inevitably confer higher risk of water loss through transpiration, particularly in scenarios of water stress. Thus fine-tuning *g_s_* for the purpose of decreasing transpiration without affecting photosynthesis rates is a breeding goal of modern agriculture. Manipulating stomatal aperture may be a useful approach to optimize plants’ water use efficiency (WUE, e.g., CO_2_ gain vs. water loss).

Changes in the osmotic potential of GCs are controlled by various ion and anion transporters localized at the plasma membrane (PM) or tonoplast (Kollist et al., 2014). PM H^+^-ATPases pumps H^+^ outside of GCs and causes hyperpolarization of the PM, resulting in K^+^ uptake through voltage-gated inward-rectifying K^+^ channels. In turn, K^+^ accumulation within GCs drives water influx, resulting in turgor increase and stomatal opening. PM H^+^-ATPases are characterized with 10 transmembrane domains and three cytosolic domains, namely the N-terminal domain, catalytic domain, and C-terminal autoinhibitory domain. The activity of PM H^+^-ATPases is directly mediated by the phosphorylation of a threonine (Thr) in the C-terminus to which 14-3-3 proteins bind, resulting in the activation of the pump (Kinoshita and Shimazaki, 1999). By contrast, the S-type anion channel (SLAC1) mediates Cl^−^ and NO_3_^−^ efflux across the GG PM, resulting in depolarization of the PM and net K^+^ efflux from GCs, and finally stomatal closure (Vahisalu et al., 2008; Munemasa et al., 2015).

**Figure 1:**
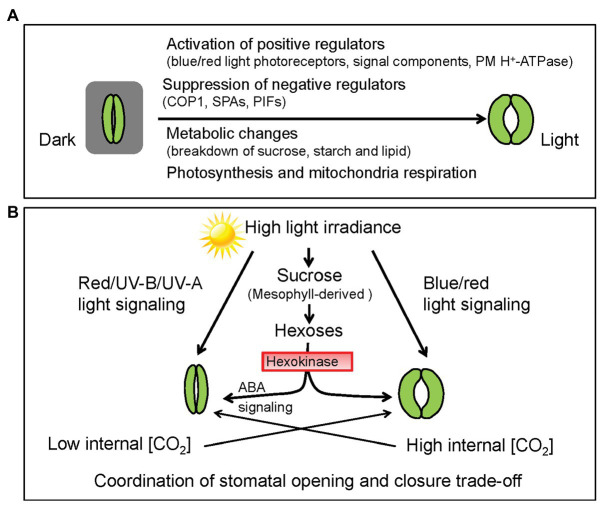
Light-mediated signaling and metabolic changes coordinate stomatal movements. **(A)** Stomatal aperture increases during dark-to-light transition. Blue and red light cooperatively induce stomatal opening through mediating different signaling pathways, yet converging on activation of the plasma membrane (PM) H^+^-ATPases in guard cells (GCs). Skotomorphogenesis regulators, such as CONSTITUTIVE PHOTOMORPHOGENIC (COP1), SUPPRESSOR OF PHYTOCHROME A (SPA), and PHYTOCHROME-INTERACTING FACTORS (PIFs) proteins, whose activities are inactivated by light, act as central regulators of stomatal closure. Besides, light-induced stomatal opening is closely related to mesophyll photosynthesis and GC-specific metabolic changes including the breakdown of sucrose, starch and lipids, which provide carbon skeletons for ATP synthesis *via* glycolysis and mitochondrial respiration. **(B)** Negative regulation of stomatal opening by red light, ultraviolet (UV-B), and UV-A signaling pathways. Mesophyll-derived sucrose acts as a signal to trigger stomatal closure in periods of high photosynthetic rate, which involves hexokinase (HXK)-mediated ABA signaling. Carbon dioxide (CO_2_) concentration [CO_2_] within GCs is constantly controlled by the stomatal aperture and photosynthetic rate. Since low [CO_2_] promotes stomatal opening while high internal [CO_2_] induces stomatal closing. The crosstalk between light‐ and CO_2_-mediated signaling deserves further investigation. Collectively, light signaling coupled with mesophyll-derived sucrose and internal [CO_2_] coordinates the trade-off between stomatal opening and closure.

Light is not only the energy source for photosynthesis but also a pivotal signal regulating multifaceted plant growth and development including seed germination, leaf development, flowering, stomatal movement, and stomatal development (Jiao et al., 2007). Advances in our understanding of light-mediated regulation of stomatal development and patterning have been recently reviewed (Wei et al., 2020). Plants experience diurnal changes in light quality and intensity that impact stomatal movement (Matthews et al., 2017). Light-induced stomatal opening can be divided into two different pathways, including the red light responses and blue light responses. Increasing evidence has been accumulated concerning the coordination of light perception, signal transduction, light-energy conversion, membrane ion transport, and metabolic changes during light-induced stomatal opening (Figure 1A; Daloso et al., 2017; Inoue and Kinoshita, 2017; Matthews et al., 2020). However, those reviews only focused on the positive effects of light on stomatal opening, neglecting the perspective that light may also trigger certain negative mechanism to prevent excessive stomatal opening (Figure 1B). Here, we discuss the dual roles of light signaling in both stomatal opening and closure, highlighting phyB-mediated signaling as a novel negative mechanism of stomatal opening. Besides, light-induced changes of metabolite levels and CO_2_ concentration within GCs also play dual roles in stomatal movement (Figure 1). The coordination of stomatal opening and closing by light is of great physiological significance for plants to maintain photosynthesis and transpiration trade-off.

## Blue and Red Light Signaling Function Synergistically to Induce Stomatal Opening

### Photoreceptors

Stomatal opening is induced predominantly by the monochromatic blue light (400–500 nm), and moderately by red (680 nm) or far-red (730 nm) light (Mao et al., 2005). It is important to note that a red light background strongly enhances stomatal responses to weak blue light, implicating that red and blue light act in concert to induce stomatal opening (Ogawa et al., 1978). The stomatal response to blue light is thought to be mesophyll photosynthesis-independent and saturates at low fluence rates (Shimazaki et al., 2007). By contrast, red light responses occur at high fluence rates and saturates at similar light intensities as mesophyll photosynthesis (Ando and Kinoshita, 2019).

Distinct wavelengths of solar irradiance are detected by multiple plant photoreceptors including the blue/ultraviolet-A absorbing cryptochromes (CRY1/2) and phototropins (PHOT1/2), red/far-red absorbing phytochromes (PhyA-E), and ultraviolet-B absorbing photoreceptor UVRESISTANCE LOCUS 8 (UVR8; Franklin and Quail, 2010). It has been well-shown that PHOT1 and PHOT2 are the major blue light-specific photoreceptors in GCs modulating stomatal movement. The *Arabidopsis phot1 phot2* loss-of-function mutants demonstrate closed stomata and attenuated response to blue light and can only respond to high fluence rates of blue light, while the single mutant has much weaker phenotype (Kinoshita et al., 2001). CRY1 and CRY2 also play roles in blue light-induced stomatal opening, acting in a PHOT-independent manner. The *cry1 cry2* double mutant has reduced stomatal aperture; however, its stomata are responsive to blue light at a very low fluence as compared to those of *phot1 phot2* double mutants (Mao et al., 2005). The stomata of high order *phot1 phot2 cry1 cry2* mutants are hardly responsive to blue light regardless of light intensities, suggesting that PHOT1/2 and CRY1/2 function synergistically to regulate stomatal opening upon blue light exposure (Mao et al., 2005). Among the five phytochrome members (phyA–E) in *Arabidopsis*, phyB was demonstrated to mediate stomatal opening under red light. The *phyB* mutants display decreased stomatal aperture grown under either white or red light compared with the wild type, while overexpression of phyB confers wider stomatal pores (Wang et al., 2010). The *phyA* mutants do not exhibit discernable difference in stomatal aperture, yet the *phyA phyB* double mutants have more severe phenotype than that of the *phyB* single mutant, indicating that phyA only functions at the absence of phyB (Wang et al., 2010). Intriguingly, it was shown that light-activated phyB generates mobile signals that move to surrounding cells and organs to trigger light responses at a distance (Kim et al., 2016), which may explain how stomata present mainly in abaxial leaf surface could respond to light coming from the top. Further studies are required to examine whether phyC, phyD, and phyE are involved in red light regulation of stomatal opening and whether they act non-cell-autonomously.

### Light Signaling Components Acting Downstream of Photoreceptors

**Figure 2:**
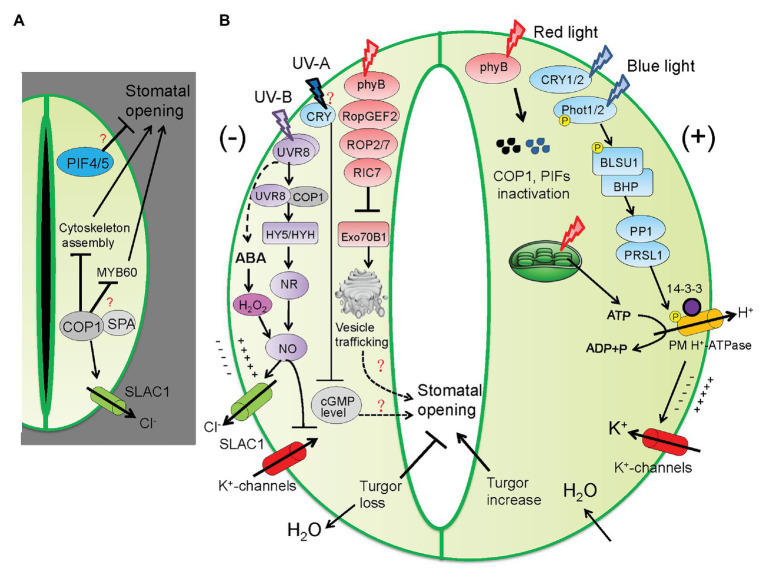
Different light signaling pathways coordinate stomatal opening and closure trade-off. **(A)** In darkness, the COP1/SPA protein complexes act in concert with PIF4/5 to suppress stomata opening. COP1 acts through disrupting microtubule organization and inhibiting S-type anion channels (SLAC1). MYB60 is a positive regulator of light-induced stomatal opening and it acts downstream of COP1 in an unidentified mechanism. **(B)** Different light signaling pathways involved in regulating stomatal opening and closure. In light or upon dark-to-light transition, different light photoreceptors act in concert to promote stomatal opening through inactivating COP1/SPA and PIFs. Blue and red light signaling converges on the activation of PM H^+^-ATPase, resulting in proton extrusion, K^+^ uptake, and ultimately water influx into GCs. Red light-activated phyB signaling also mediates a brake mechanism comprised of the ROPGEF2-ROP2/7-RIC7 signal cascade to suppress stomatal opening. RIC7 suppresses the activity of Exo70B1, a component of the vesicle trafficking machinery. UV-B and UV-A irradiance prevents blue/red light-mediated stomatal opening. UV-B promotes the production of abscisic acid (ABA) and in turn hydrogen peroxide (H_2_O_2_) and nitric oxide (NO). In parallel, UV-B perception by the UVR8 dimer induces the interaction of UVR8 monomer with COP1 in the nucleus, resulting in upregulated expression of *ELONGATED HYPOCOTYL5* (*HY5*) and *HY5 HOMOLOG* (*HYH*) that stimulate NO production. NO stimulates stomatal closure through activating SLAC1 and inhibiting K^+^ channels. UV-A induces decrease in the level of cyclic guanosine monophosphate (cGMP) dependent on the cGMP phosphodiesterase (AtCN-PDE1). phyB, phytochrome B; CRY, cryptochrome; PHOT, phototropin; BLUS1, BLUE LIGHT SIGNALING 1; BHP, BLUE LIGHT-DEPENDENT H^+^-ATPASE PHOSPHORYLATION; PP1, PROTEIN PHOSPHATASE 1; PRSL1, PP1 REGULATORY SUBUNITS2-LIKE PROTEIN 1; SLAC1, SLOW ANION CHANNEL 1; ROP, Plant Rho-type GTPase; ROPGEF2, ROP GUANINE NUCLEOTIDE EXCHANGE FACTOR 2; RIC7, ROP-interactive Cdc42‐ and Rac-interactive binding motif-containing protein 7. Arrow and bar-ended lines represent activation and inhibition, respectively. Dotted lines denote indirect regulation. Question mark denotes unclear mechanism.

Different photoreceptors share both common and distinct components of signaling pathways. The most eminent light signaling protein functioning downstream of PhyB and CRY1/2 is CONSTITUTIVE PHOTOMORPHOGENIC (COP1), a RING-finger-type E3 ubiquitin ligase that accumulates in darkness and represses seedling photomorphogenesis (Wang et al., 2001; Yang et al., 2001). The dark-grown *cop1* loss-of-function mutants display constitutive open stomata that resemble those of light-grown plants, providing convincing evidence that COP1 acts as a central suppressor of stomatal opening (Mao et al., 2005; Wang et al., 2010). Further investigation established the link between COP1 activity with the regulation of S-type anion channels and cytoskeletal organization (Khanna et al., 2014). The dark-grown *cop1* mutants exhibited impaired activity of SLAC1 as well as stabilization of microtubule arrays within GCs, which are collectively accountable for the constitutive open stomata in dark (Khanna et al., 2014). Given that COP1 activity is promoted by WD40 domain-containing PHYTOCHROME A SUPRESSOR (SPA) proteins through forming protein complexes (Laubinger et al., 2004; Hoecker, 2005), it is speculated that COP1 acts together with SPA proteins to maintain stomatal closure in dark. In support of this notion, the *spa1 spa2 spa3* triple mutants demonstrate open stomata in dark, resembling those of the *cop1* mutants (Wang et al., 2010). Interestingly, it was shown that the expression of *MYB60*, a GC-specific transcription factor regulating light-induced stomatal opening, is enhanced by overexpression of *PHYB* or mutation of *COP1* (Cominelli et al., 2005; Wang et al., 2010), suggesting that MYB60 functions downstream of COP1. Further study is required to elucidate how COP1 regulates the expression or protein activity of MYB60 and whether SPA proteins regulate stomatal closure through pathways independent of COP1. In addition, PHYTOCHROME-INTERACTING FACTORS (PIFs), belonging to basic helix-loop-helix (bHLH) transcription factor family, may also function as negative regulators of stomatal opening, as the *pif4 pif5* double mutants exhibit increased stomatal aperture (Wang et al., 2010). However, the molecular mechanisms underlying PIF-mediated stomatal closure remain unexplored (Figure 2A). Collectively, during dark-to-light transition, different photoreceptors act in concert to inactivate those negative regulators of photomorphogenesis, including translocation of COP1 from the nucleus to the cytoplasm, destabilization of the COP1-SPA complexes, phosphorylation and degradation of PIFs, consequently contributing to stomatal opening.

In addition, accumulating evidence shows that blue and red light signaling promotes stomatal opening through converging on the phosphorylation of the PM H^+^-ATPases. The signaling intermediates underlying blue light-mediated activation of H^+^-ATPases have been elucidated in recent years. Blue light receptors PHOT1/2, activated through auto-phosphorylation, trigger phosphorylation of the protein kinase BLUE LIGHT SIGNALING 1 (BLUS1). BLUS1 physically interacts with the Raf-like protein kinase, BLUE LIGHT-DEPENDENT H^+^-ATPASE PHOSPHORYLATION (BHP), forming early signaling complexes with PHOT1/2 to transmit the signal toward the protein phosphatase 1 (PP1) and its regulatory subunit PRSL1, ultimately resulting in the phosphorylation of H^+^-ATPase to which 14-3-3 protein binds (Takemiya et al., 2006, 2013a,b; Takemiya and Shimazaki, 2016; Hayashi et al., 2017; Figure 2B). However, it is not known regarding the substrates for protein kinases BLUS1, BHP and PP1 in GCs, and the protein kinase mediating direct phosphorylation of H^+^-ATPases, which require further analyses.

It was also shown that red light resulted in the accumulation of K^+^ in GCs, indicating the activation of PM H^+^-ATPases (Olsen et al., 2002). Yet red light-induced phosphorylation of H^+^-ATPases in GCs was not observed in trials due to lacking of proper experimental approaches (Kinoshita and Hayashi, 2011; Suetsugu et al., 2014). Ando and Kinoshita (2019) developed an immunohistochemical technique using whole leaves instead of the isolated epidermis and successfully detected the activation of PM H^+^-ATPases upon red light illumination. Similar with the effects of blue light, red light confers the phosphorylation of the penultimate C-terminal residue Thr of PM H^+^-ATPase in whole leaves (Ando and Kinoshita, 2019). Furthermore, the treatment of photosynthetic electron transport inhibitor 3‐ (3,4-dichlorophenyl)-1,1-dimethylurea (DCMU) inhibits the phosphorylation of H^+^-ATPases and light-induced stomatal opening, supporting that photosynthesis by both mesophyll cells and GCs is indispensable for red light-induced activation of PM H^+^-ATPases (Wang et al., 2014; Azoulay-Shemer et al., 2015; Ando and Kinoshita, 2019). It is postulated that certain photosynthetic products act as signals contributing to red light-induced phosphorylation of PM H^+^-ATPase of GCs, a hypothesis that needs to be clarified. Interestingly, it was shown that red light-induced phosphorylation of PM H^+^-ATPase is saturated at 50 μmol m^−2^ s^−1^, and the phosphorylation level no longer increases even the light intensity reaches up to 600 μmol m^−2^ s^−1^ (Ando and Kinoshita, 2019). Taken together, blue and red light promote stomatal opening through employing different mechanisms that are PHOT-mediated signaling and mesophyll photosynthesis-dependent, respectively. Different light signaling pathways converge on the phosphorylation and activation of PM H^+^-ATPases, resulting in successive procedures including proton extrusion, K^+^ uptake, water influx into GCs, and consequent stomatal opening.

## Negative Regulation of Stomatal Opening by Red Light Signaling

Light-induced stomatal opening facilitates the CO_2_ uptake necessary for photosynthesis; however, extravagant stomatal opening may lead to excessive water loss and death threat. Therefore, it is not surprising that plants have evolved a pacing mechanism that limits stomatal opening in response to light (Figure 2B). To date, several negative regulators of light-mediated stomatal opening have been characterized. Two Rho-type (ROP) GTPases (ROP2 and ROP7) act redundantly to suppress red light-induced stomatal opening, given that the *rop2 rop7* loss-of-function mutants showed much larger stomatal pores than the *rop2* or *rop7* single mutant under red light but not under blue light (Jeon et al., 2008; Hwang et al., 2011). It is well-known that activities of ROP proteins are enhanced by their interacting partners named ROP GUANINE NUCLEOTIDE EXCHANGE FACTORs (ROPGEFs; Oda and Fukuda, 2012). In this context, ROPGEF2 and ROPGEF4 were shown to physically interact with ROP7. Similar with the stomatal phenotype of *rop7*, the *ropgef2 ropgef4* double mutants exhibited wider stomata aperture (increased *gs*), suggesting that RopGEF2 and RopGEF4 also function as negative regulators of stomatal opening upon white light illumination (Wang et al., 2017). The most significant finding is that the RopGEF2/4-ROP2/7 module acts genetically downstream of phyB to suppress light-induced stomatal opening based on several lines of evidence. First, the *phyB ropgef2 ropgef4* triple mutants displayed similar phenotype as the *ropgef2 ropgef4* double mutant under white light. Second, phyB directly interacts with RopGEF2 in both light and dark conditions. Third, upon red light irradiation, the active Pfr form of phyB in the cytoplasm, before translocation into the nucleus, enhance the activities of RopGEF2 toward both ROP2 and ROP7, and therefore, mediating stomatal closure (Wang et al., 2017). Additionally, it was shown that activated ROP proteins associate with the ROP-interactive Cdc42‐ and Rac-interactive binding motif-containing protein 7 (RIC7) to modulate downstream processes (Wu et al., 2001; Gu et al., 2005), indicating that RIC7 negatively regulates stomatal opening. Indeed, light-induced stomatal opening was enhanced in the *ric7* background, whereas it was repressed in *RIC7* overexpression lines (Hong et al., 2016). Through yeast-two-hybrid screening, the exocyst subunit Exo70 family protein B1 (Exo70B1), a component of the vesicle trafficking machinery, was identified as a target protein of RIC7. Both the *exo70b1* single mutant and the *ric7 exo70b1* double mutant demonstrated attenuated stomatal opening in response to light, indicating that Exo70B1 stimulates light-induced stomatal opening *via* a yet unidentified mechanism and its activity is inhibited by RIC7 (Hong et al., 2016).

To sum up, photo-activated phyB enhances the RopGEF2/4-ROP2-RIC7 signaling pathway to suppress the activity of Exo70B1, and consequently to negatively modulate stomatal opening in red light-dependent manner (Wang et al., 2017). This phyB-mediated brake mechanism is particularly beneficial in scenarios of high light irradiance, allowing fine-tuning of stomatal aperture to optimize CO_2_ uptake and water loss. Yet it remains to be shown which domain of phyB is responsible for its interaction with RopGEF2, and which domain is required for the activation on RopGEF2, and whether phyB-mediated activation of the RopGEF2/4-ROP2 module is associated with increasing red light intensity. Further, the physiological significance of the red light-mediated negative regulation of stomatal opening needs to be unraveled. It is interesting to examine whether the negative regulation by red light is directly linked to prevent excessive stomatal opening.

## Ultraviolet Light Signaling Inhibits Blue and Red Light-Induced Stomatal Opening

UV-B radiation (280–315 nm) is a part of the solar radiation, representing about 7% of the electromagnetic radiation emitted from the sun (Tossi et al., 2014). Generally the intensity peak of UV-B light is closely correlated with that of blue and red light, during which stomata tend to be widely open and photosynthesis is most active. It is widely assumed that UV-B light signaling operates as a surveillance mechanism to prevent excessive stomatal opening and thus reduce water loss by transpiration (Figure 2B). The UV-B light photoreceptor UVR8 mediates a specific UV-B signaling pathway that regulates diverse growth and development processes, as well as self-protecting responses, such as downward leaf curling and stomatal closure (Yin and Ulm, 2017). The mechanisms underpinning UV-B light-mediated stomatal closure have been extensively studied. First, UV-B induces the production and accumulation of abscisic acid (ABA), hydrogen peroxide (H_2_O_2_), and nitric oxide (NO), which are all involved in promoting stomatal closure (Tossi et al., 2012, 2014). In parallel, UV-B perception by the UVR8 dimer induces the interaction of UVR8 monomer with COP1 in the nucleus, resulting in upregulated expression of *ELONGATED HYPOCOTYL5* (*HY5*) and *HY5 HOMOLOG* (*HYH*; Huang et al., 2012). HY5 and HYH are known to promote NO generation through positively modulating the expression and activity of nitrate reductase (NR; Jonassen et al., 2008, 2009). NO plays a central role in regulating stomatal closure through enhancing the activity of anion channels (SLAC1) and suppressing the activity of inward-rectifying K^+^ channels (Garcia-Mata et al., 2003; Zhao et al., 2012; Tossi et al., 2014). It is worth noting that NO is able to inhibit blue light-induced stomatal opening (Zhao et al., 2012), implicating that UV-B light is able to antagonize blue light-induced stomatal opening.

In contrast to the well-reported effects of UV-B light, much less is known about the roles of UV-A radiation (315–400 nm) on stomatal movement. Recently, it was shown that *Arabidopsis* epidermis illuminated with UV-A in combination with blue and red light demonstrated greatly decreased stomatal aperture than those illuminated with only blue and red light, and that the effect was strong at 3 h post exposure and decreases with time (Isner et al., 2019). It is noted that UV-A irradiation could not trigger closure in stomata that had previously been opened by blue and red light illumination. In addition, it was revealed that a decreased level of cytosolic cyclic guanosine monophosphate (cGMP) in guard and mesophyll cells was underlying UV-A-mediated suppression of blue‐ and red-light-induced stomatal opening. Isner et al. further investigated the physiological significance of long-term UV-A irradiation on plant growth. Interestingly, they found that UV-A promotes plant growth and increase transpirational water loss but decreases the overall WUE. Taken together, UV-B and UV-A light signaling acts in concert to limit stomatal opening in periods of strong solar irradiance. Although it was shown that the photoreceptor UVR8 mediates the perception of both UV-B and UV-A wavelengths (Rai et al., 2020), UV-A-mediated stomatal closure does not require UVR8 (Isner et al., 2019). Considering that CRY1 and CRY2 also act as photoreceptors for UVA radiation (Lin et al., 1995), further studies are needed to elucidate how CRY1/2 integrate blue and UV-A light signals to regulate stomatal responses and how decreased cGMP levels mediate the inhibition of blue/red light-induced stomatal opening.

## Light-Induced Metabolic Changes in Guard Cells Stimulate Stomatal Opening

**Figure 3:**
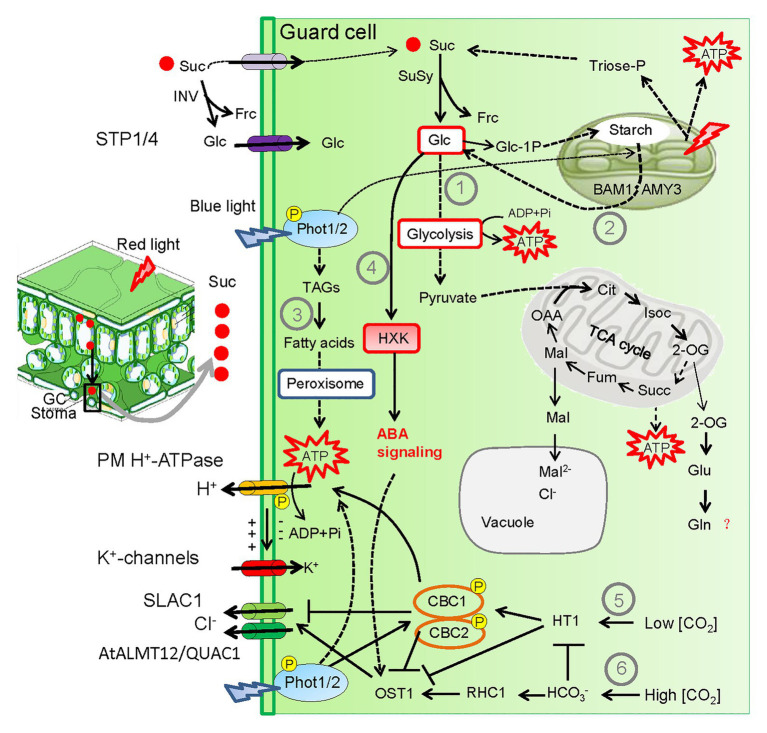
Light-induced metabolic changes and CO_2_ concentration [CO_2_] within GCs coordinate stomatal opening and closure trade-off. Light-induced stomatal opening are tightly associated with GC metabolic changes including the breakdown of sucrose, starch, and triacylglycerols (TAGs; ①–③) to provide carbon skeletons for ATP synthesis *via* glycolysis and mitochondrial metabolism. Particularly, degradation of TAGs and starch are dependent on blue light signaling. Glucose (Glc) is the major intermediate of starch degradation. In periods of strong light intensity, mesophyll-derived sucrose (Suc) is translocated to the apoplastic space by transpirational stream. Suc is either transported into the GC or cleaved by inverse (INV) in the apoplast to produce Glc and fructose (Frc). Then Glc is uptaken into GCs *via* the monosaccharide/proton symporters Sugar Transport Protein 1/4 (STP 1/4). Within the GC, sucrose-derived Glc stimulates stomatal closure through hexokinase (HXK)-induced ABA signaling under carbon-replete conditions (④). In addition, CONVERGENCE OF BLUE LIGHT AND CO_2_ 1 (CBC1) and CBC2 that act as positive regulators of blue light‐ and low CO_2_ concentration [CO_2_]-mediated stomatal opening through inactivating the SLAC1 (⑤). By contrast, high [CO_2_] signaling activates OPEN STOMATA1 (OST1), a well-established component of ABA signaling to bring about stomatal closure through activating SLAC1 and the R-type AtALUMINUM ACTIVATED MALATE TRANSPORTER 12/QUICK ANION CHANNEL 1 (AtALMT12/QUAC1) channels (⑥). Thus [CO_2_] within GCs provides another layer of mechanisms coordinating stomatal opening and closure. Arrow and bar-ended lines represent activation and inhibition, respectively. Dotted lines denote indirect regulation or involvement of several steps. Question mark denotes unclear mechanism. SuSy, sucrose synthase; TCA, tricarboxylic acid; Cit, citrate; IsoC, isocitrate; 2-OG, 2-oxoglutarate; Succ, succinate; Fum, fumarate; Mal, Malate; OAA, oxaloacetate; Glu, glutamate; Gln, glutamine.

Stomatal opening is an active, energy-demanding process, as H^+^-ATPase consumes adenosine triphosphate (ATP) to pump proton out of the GC cytoplasm. It has been well elucidated in recent years that light-induced stomatal opening is tightly associated with the GC-specific metabolic changes including the breakdown of sucrose, starch, and lipids to provide carbon skeletons for ATP synthesis *via* glycolysis and mitochondrial metabolism and to produce osmolytes such as sugars and malate (Figure 3; Antunes et al., 2012; Daloso et al., 2015; Horrer et al., 2016; Mclachlan et al., 2016; Medeiros et al., 2018; Flütsch et al., 2020a). GC chloroplasts contain the entire photosynthetic machinery that produces sucrose and ATP by the chloroplast electron transport chain upon exposure to light, although the photosynthesis capacity of GCs is much lower compared with that of mesophyll cells (Suetsugu et al., 2014). Besides, GCs have highly specialized mitochondrial metabolism with higher activity of mitochondrial enzymes compared with mesophyll cells (Daloso et al., 2017). During light and K^+^-induced stomatal opening, sucrose synthase (SUS) and invertase (INV) in GCs convert sucrose into hexoses (glucose and fructose), which are used as substrates for glycolysis and mitochondrial respiration (Antunes et al., 2012; Daloso et al., 2015). Interestingly, transgenic *Nicotiana tabacum* plants with GC-specific overexpression of *SUS3* exhibited increased stomatal aperture (higher *gs*) and differential accumulation of citrate, succinate, and fumarate, which are important intermediates of the tricarboxylic acid (TCA) cycle (Daloso et al., 2016). Medeiros et al. (2018) further corroborated that light-activated glycolysis and TCA cycle was associated with stomatal opening using techniques of ^13^C-sucrose isotope kinetic labeling and metabolomic analysis. They found that sucrose breakdown provides carbon skeletons for glutamine (Gln) biosynthesis, which may in turn activate certain downstream pathways to support light-mediated stomatal opening (Medeiros et al., 2018). Although the direct evidence concerning the exact role of Gln during light-induced stomatal opening is lacking, it is suggested that Gln may enhance activities of fumarase (FUM) isoforms that convert malate into fumarate, which is an important organic acid involved in stomatal movement (Lima et al., 2018). Future studies using system biology approaches are warranted to elucidate the physiological relevance of sucrose breakdown toward Gln synthesis during light-mediated stomatal opening.

It was also shown that starch is rapidly degraded in response to light, concomitant with light-induced stomatal opening (Horrer et al., 2016). Interestingly, starch in GCs is accumulated at the end of the night, while starch in mesophyll cells is degraded during the nighttime (Graf et al., 2010). Further analysis showed that β-amylase 1 (BAM1) and α-amylase 3 (AMY3) enzymes are specifically required for light-induced starch degradation. Simultaneous mutation of *BAM1* and *AMY3* resulted in significantly decrease in stomatal aperture and retarded plant growth, particularly under high light intensities. It is worth mentioning that starch degradation is specifically triggered by blue light, whereas red light triggers photosynthesis and starch synthesis. This notion is corroborated by the observation that starch degradation is impaired in blue light signaling mutants such as *phot1/2* and *blsu1* (Horrer et al., 2016). Besides, the activation of PM H^+^-ATPase is a prerequisite for starch breakdown, given that starch breakdown is defective in the *aha1* mutants with disrupted proton pumping (Horrer et al., 2016). It was previously thought that carbon skeletons released from starch breakdown within the GCs were used to synthesize malate (Mal), an important counter-ion for K^+^ in the vacuole allowing water uptake and a signaling molecule in the GC cytosol (Horrer et al., 2016). Yet it was recently shown that the Mal content in isolated GCs was not different between the wild type and the *amy3 bam1* mutant defective in starch degradation after blue light treatment, suggesting that Mal is not the major intermediate of starch degradation (Flütsch et al., 2020a). Strikingly, glucose (Glc) content was significantly decreased in GCs of the *amy3 bam1* mutant after blue light treatment compared with the WT, indicating that Glc is the primary starch-derived metabolite in GCs. Glc can be used as carbon sources to synthesize pyruvate *via* glycolysis, which then enters the TCA cycle to furnish ATP for H^+^ pumping (Flütsch et al., 2020a). In addition, it has been shown that mesophyll-derived Glc can be transported into GCs *via* the monosaccharide/proton symporters Sugar Transport Protein 1 (STP1) and STP4 that are localized at the PM of GCs. The *stp1 stp4* mutant shows reduced levels of Glc and almost lacking of starch in GCs after white light illumination (Flütsch et al., 2020b). Collectively, Glc, either uptaken into GCs from mesophyll cells *via* STP transporters, or derived from blue light-induced starch degradation within GCs, contributes to rapid stomatal opening in response to light, highlighting the coordination of mesophyll and GC carbohydrate metabolism, and the tight connection between stomatal movement and photosynthesis.

In addition, it was shown that the concentration of triacylglycerols (TAGs) and lipid droplets in GCs is reduced during dark-to-light transition (Mclachlan et al., 2016). Light-mediated stomatal opening is retarded in mutants with defects in TAG catabolism as well as in the *phot1 phot2* double mutants, supporting that TAG breakdown is dependent on blue light signaling (Mclachlan et al., 2016). It is suggested that TAGs are used as a vital energy source, stimulating ATP and malate production through β-oxidation and TCA cycle (Vavasseur and Raghavendra, 2005; Daloso et al., 2017). Furthermore, a recent study systematically examined red light-induced metabolic changes in GCs of *Arabidopsis*, showing that red light-induced stomatal opening is correlated with decreased contents of ABA and jasmonic acid (JA) in GCs in addition to TCA cycle and carbon homeostasis (Zhu et al., 2020). It will be interesting to investigate how light and phytohormone signaling cross-talk in regulating stomatal movement.

## Mesophyll-Derived Sucrose Coordinates the Trade-Off Between Stomatal Opening and Closure

In phloem loader species such as *Arabidopsis*, sucrose is a major form of photoassimilates transported from mesophyll cells to the apoplastic space of GCs *via* transpirational stream. The amount of sucrose accumulated in the GC apoplast is correlated with the width of stomatal aperture (Lu et al., 1995). As discussed above, light-induced sucrose breakdown within GCs supports stomatal opening. Interestingly, sucrose has been defined as a negative regulator of stomatal opening in periods of high photosynthetic rate (Lima et al., 2018). Indeed, sucrose-induced stomatal closure is a highly conserved mechanism in different plant species regardless of different photosynthetic machineries [C_3_, C_4,_ or crassulacean acid metabolism (CAM)] and phloem-loading strategies (symplastic or apoplastic; Kottapalli et al., 2018).

The osmotic properties of sucrose has for a long time been believed to confer stomatal closure, as the accumulation of mesophyll-derived sucrose in the apoplastic space renders the water potential of the apoplast lower than that of the cytosol, resulting in the efflux of water from GCs and subsequent stomatal closure (Kang et al., 2007). However, recent studies overthrew the idea of osmotic effects of sucrose based on several lines of evidence. First, application of sucrose was able to induce stomatal closure in various plant species, including *Arabidopsis*, tomato, and soybean (Kelly et al., 2013; Li et al., 2016; Kottapalli et al., 2018), whereas application of mannitol (an osmotic control) did not cause stomatal closure (Kelly et al., 2013; Medeiros et al., 2018). Second, it was shown that exogenous addition of sucrose triggered the production of NO, an important component of ABA signaling (Kelly et al., 2013). Third, sucrose-induced stomatal closure was prevented by an inhibitor for hexokinase (HXK, a hexose phosphorylating enzyme), providing compelling evidence that HXK-induced ABA signaling is underpinning sucrose-mediated stomatal closure (Kelly et al., 2013). The function of HXK in stomatal movement is further corroborated by the observation that overexpression of the *Arabidopsis* HXK1 in GCs resulted in high *g_s_* at low light intensity yet low *g_s_* at high light intensity (Lugassi et al., 2015). Taken together, sucrose accumulation in periods of high photosynthetic rate reduces *g_s_*, highlighting the significance of mesophyll-derived sucrose as a metabolic signal to coordinate photosynthesis and transpiration. Specifically, sucrose-derived hexoses are phosphorylated by HXK, leading to either stomatal opening or closure according to the concentration of sucrose/hexoses within GCs and the ambient light intensity (Kelly et al., 2013; Lugassi et al., 2015). In addition, it was shown that ATP affects stomatal aperture in a dose-dependent manner, as low (<35 uM) and high (>150 uM) concentration of ATP caused stomatal opening and closure, respectively (Clark et al., 2011). It is worth mentioning that mitochondrial respiration in mesophyll cells is downregulated by light, while respiration in GCs is likely unaffected by light (Daloso et al., 2015, 2016). Thus, we speculate that under high light irradiance, excessive ATP production *via* photosynthetic activity and mitochondria metabolism within GCs may also contribute to decreasing stomatal aperture. This hypothesis needs to be experimentally verified.

## Cross-Talk Between Light and Co_2_ Signaling Represents Another Layer of Mechanisms Coordinating Stomatal Opening and Closure

Guard cells must respond to constantly changing environment factors, such as light (quantity and quality) and CO_2_ concentration [CO_2_], and adjust stomatal aperture accordingly. It has been well shown that low [CO_2_] stimulates stomatal opening, while high [CO_2_] induces stomatal closure (He et al., 2018). Indeed, a change in the intercellular [CO_2_] (*C_i_*) might act as a mesophyll-to-GC transmissible signal mediating stomatal behavior (Wong et al., 1978; Huxman and Monson, 2003). Perhaps one of the most significant findings is the convergence of blue light and CO_2_ signaling pathways regulating stomatal movement (Hiyama et al., 2017). Given that light decreases *C_i_* due to photosynthetic CO_2_ fixation, thus, light and low *C_i_* synergistically stimulate stomatal opening to maximize CO_2_ uptake. Blue light induce phosphorylation of CONVERGENCE OF BLUE LIGHT AND CO_2_ 1 (CBC1) and CBC2 protein kinases in a PHOT-dependent manner. CBC1 and CBC2 act redundantly as positive regulators of stomatal opening through inactivating the S-type anion channel (SLAC1). CBC1 and CBC2 act in the same pathway as HIGH LEAF TEMPERATURE 1 (HT1), a RAF-like MAP kinase whose activity is activated in response to low *C_i_* (Hashimoto et al., 2006). HT1 physically interacts with and phosphorylates CBC1/CBC2. Thus, CBC1 and CBC2 act as convergence points of the blue light and CO_2_ signaling pathways mediating stomatal opening (Hiyama et al., 2017).

Whereas in scenarios of high [CO_2_], bicarbonate (HCO_3_^−^) acts as the intracellular signal to activate OPEN STOMATA1 (OST1) and to relieve HT1-CBC1/2-mediated suppression of OST1 (Chater et al., 2015; Hsu et al., 2018). OST1, a member of SNF-related protein kinases (SnRK2s), is an important component of ABA signaling that has been well-characterized to stimulate stomatal closure through activating both slow-type SLAC1 and rapid-type AtALUMINUM ACTIVATED MALATE TRANSPORTER 12/QUICK ANION CHANNEL 1 (AtALMT12/QUAC1) anion efflux channels (Mustilli et al., 2002; Imes et al., 2013). Collectively, high *C_i_* activates SLAC1, resulting in ion efflux from GCs and stomatal closure (Zhang et al., 2018). Therefore, we speculate that in periods when stomata are most widely open, elevated [CO_2_] within the intracellular space of two GCs and the underlying mesophyll cells may trigger closing of stomata, providing a feed-forward mechanism to limit water evaporation from leaves. However, a recent study also showed that CBC1/CBC2 may negatively regulate stomatal opening through inactivating H^+^-ATPase, given that the *cbc1 cbc2* double mutant exhibited wider stomatal aperture under both dark and blue light conditions and that the level of phosphorylation of C-terminal Thr of H^+^-ATPase was higher in double mutant GCs (Hayashi et al., 2020). Therefore, CBC1 and CBC2 play dual roles in stomatal movement, which is in accord with the dual functions of [CO_2_], i.e., low [CO_2_] promotes stomatal opening while high [CO_2_] inhibits stomatal opening. Considering the tight link between *C_i_* and photosynthesis, the CO_2_ signaling coupled with sucrose signaling within GCs or transmitted from mesophyll cells are important mechanisms regulating both stomatal opening and closure.

## Conclusion and Perspectives

Accumulating evidence unequivocally demonstrates that photomorphogenesis regulators act as positive regulators of stomatal opening, while skotomorphogenesis regulators act as suppressors of stomatal opening. In this review, we summarized recent advances concerning the positive and/or negative regulation by different light (blue, red, UV-B, and UV-A) signaling and light-induced changes in metabolite levels and [CO_2_] within GCs fine-tune stomatal aperture. In darkness, skotomorphogenesis regulators, such as COP1, SPAs and PIFs, play central roles in the control of stomatal closure. The mode of action of PIF-mediated stomatal closure needs to be elucidated. Upon dark-to-light transition, blue and red light photoreceptors act in concert to suppress those negative regulators and activate downstream signal cascades that converge on the activation of PM H^+^-ATPase, resulting in stomatal opening. It has also been shown that PhyB-mediated red light signaling, coupled with UV-B and UV-A light signaling, may operate as a “brake” to negatively regulate stomatal opening. In addition, light-induced GC-specific metabolic changes play pivotal roles in promoting stomatal opening, while mesophyll-derived carbohydrates and high [CO_2_] may trigger negative regulation mechanisms. These findings suggest that in periods of high light intensity (e.g., strong solar irradiance at noon), plants have to elaborately control stomatal opening and closure in order to coordinate carbon gain and water loss.

Yet several questions remain unresolved. For instance, whether the negative regulation of stomatal opening by red light is linked to prevent excessive stomatal opening? In order to investigate the physiological significance of red light irradiance, future studies are needed to define the positive and negative effects of red light in terms of light intensity and/or illumination time. Second, how different light signaling interacts in regulating the trade-off between stomatal opening and closure in the natural environment? To gain an insight into the physiological significance of different light interactions, it is imperative to measure the red, far-red, blue, UV-B, and UV-A spectra of solar irradiation during the course of a day and examine the possible association between different wavelength ratios with the size of stomatal pore and WUE. Furthermore, an integrated systemic approach to monitor daily changes of ambient and internal [CO_2_], the transcriptomic, proteomic, and metabolic alterations in GCs may lead to a comprehensive picture of signal integration in stomatal opening and closure trade-off. Understanding stomatal physiology in response to environmental factors, such as light and CO_2_, is crucial for controlling plants’ photosynthetic rates and water status. The crosstalk between light – and CO_2_-mediated signaling deserves further investigation.

Cereal grasses such as rice, maize, and wheat provide the majority of human nutrition and are important for global food security, and the stomatal complexes in grasses differ greatly to that of *Arabidopsis*, which are comprised of dumbbell-shaped GCs flanked by subsidiary cells. However, our knowledge regarding light-mediated regulation of stomatal movement in grasses is very limited, which is an interesting avenue worthy of further investigation. Knowledge on the mechanisms underlying light-regulated stomatal opening and closure may have potential applications toward agricultural improvement.

## Author Contributions

HW conceived the topic. JY, CL, and HW wrote the manuscript and drew the figures. DK and FG edited its final form. All authors contributed to the article and approved the submitted version.

### Conflict of Interest

The authors declare that the research was conducted in the absence of any commercial or financial relationships that could be construed as a potential conflict of interest.
